# On social psychopathology: Example with German justice

**DOI:** 10.1192/j.eurpsy.2021.2042

**Published:** 2021-08-13

**Authors:** M. Michailov, E. Neu, U. Welscher, A. Gerdzhikov, J. Foltinova, V. Foltin, M. Holler, G. Weber

**Affiliations:** 1 Pharmaco-physiology, Inst. Umweltmedizin (IUM) c/o ICSD/IAS e.V., POB 340316, 80100 M. (Int.Council Sci.Develop./Int.Acad.Sci. Berlin-Innsbruck-Muenchen-NewDelhi-Paris-Sofia-Vienna), Muenchen, Germany; 2 Fac. Medicine, Univ. Bratislava, Bratislava, Slovak Republic; 3 Of Health & Social Work, St. Elizabeth Univ., Bratislava, Slovak Republic; 4 Fac. Economics (dean), Univ. Hamburg, Hamburg, Germany; 5 Fac. Psychology (dean), Univ. Luxemburg & Vienna, Vienna, Austria

**Keywords:** psychopathological justice, UNO-Agenda 21, social psychiatry

## Abstract

**Introduction:**

INTRODUCTION-OBJECTIVES. Similar to philosophy (regina-scientiarum) is psychiatry fundamental-discipline for all-medical&social sciences. Immanuel KANT: Primus inter pares of ARISTOTELES&PLATON considered over 200years ago physiological and pragmatic anthropology-[1]. Social physiology is given-[3-4]. Consideration of social-psychopathology in German-justice-[2].

**Objectives:**

REFERENCES. [1]-Kant,I: BdXI,371-393, BdXII,399,625-638:Suhrkamp-TB-Wiss. [2]-Neu,E/Michailov, M.Ch/Welscher,U/et-al.: 2a.-FISP-2018-Beijing/Philos (1348-50,1373-4,1420); 2013-Athens Abstr.Book(AB):464-5/503-4/766; 2008-Seoul-ProcVol.4: 101-108/195-214/229-237; 2003-Istanbul:273-281; IVR-2019-Luzern (Law), Progr-Book p.116. 2b.-EPA-2020-Madrid, Eur.Psychiatry 63S, EPP0834/5+EPV0581/1470; EPA-2019-Warsaw, 56S,S689; EPA-2018-Nice, 48/S1, S623&567&662. 2c.-WPA-2021-Bangkok (in-press). 2019-Lisbon, E-Poster WCP19-2137/-1822/-1839. 2018-Mexico-City, Abs.-Book WCP18-0584/-0625/-0643/-0654. 2011-Buenos-Aires, AB:PO1.200. [3]-Glasachev,O: Sechenov Physiol.J 80/no5, 1994,p.139-143 (Russian), ref. in English. [4]-Seeley,T.D: Social-Physiology Honey-Bee, Book-1996.

**Methods:**

[5]-Daily-journal-“tz”-München, esp. every Tuesday 2016-2019: reports on Res.-Houses,e.g. 14.02.2019, 15.02.17, 06.12.16/p.10, 18.10.16/p.10, 17.11.2020/p.6. Süddt.Zeitung-München no172/p.30,2017. Mü.-Merkur:16.11.2020/p.32; 19.11.2020/p.29. FAZ:20.10.2019/p.53; 16.11.2020/p.21. BUROW,P: Justiz am Abgrund&Ein Richter klagt an. GNISA,J, Präs.-Dt.Richterverein: „Ende der Gerechtigkeit“, Herder-2017. SCHLEIF,T/Amtsrichter: Buch „Urteil: ungerecht“, zeit-online 24.10.2019. Hans-Jochen&Liselotte VOGEL:„Mehr Gerechtigkeit“, 2019 „Wohn-Irrsinn“(Enteignungen). ZANTKE,S (Richter-Amtsgericht-Zwickau): TV-Programm„Auf einen Blick“ Nr.47,2018,S.24. [6]-Luetge,Ch et-al.(ed): Experimental-Ethics, Palgrave-Macmillan 2014. [7]-Pegoraro,R/Vatican: «Arzt&Christ» 38:3-55,1992.

**Results:**

RESULTS Prominent German experts for justice: Patrick BUROW, Jens GNISA/President Law-Association/Germany, Torsten SCHLEIF/Amtsrichter, Hans-Jochen VOGEL/Ex-Minister, Stephan ZANTKE/Richter reflect in their books fundamental-criticism of German justice [5]. Inst.-Ecol.-Med./IUM investigated psychopathology of juridical-offices&law-court in Munich (Amtsgericht). Analysis suggests presence of symptoms for pseudologia-phantastica, psychopathy, cyclophrenia (esp.mania),etc. conc. observations on many persons (n>30).

**Conclusions:**

CONCLUSION. Juridical situation in Germany demonstrates contradiction to human-rights (EU-CHARTA, art.1-8/25-26/33-35), ignoring moral-philosophy, related to human obligations/I.Kant-[1], experimental ethics/Ch.Luetge et-al.-[6], medical personnel/R.Pegoraro-[7]. Only paradigm change in law-policy incl. enlarged implication of moral philosophy-theology, psychiatry-psychology, social philosophy in juridical eduction & practices could counteract disastrous juridical situation in Germany and on global level, supporting UNO-AGENDA21 for better education-health-ecology-economy (see 2.).

**Disclosure:**

No significant relationships.

